# Controversial Concepts Regarding T Helper 1 and T Helper 2 Cytokines in Hepatitis C Virus Infected Patients

**DOI:** 10.5812/hepatmon.8067

**Published:** 2013-05-21

**Authors:** Mojgan Noroozi Karimabad, Gholamhossein Hassanshahi, Mohammad Kazemi Arababadi

**Affiliations:** 1 Molecular Medicine Research Center, Rafsanjan University of Medical Sciences, Rafsanjan, IR Iran; 2 Immunology of Infectious Disease Research Center, Rafsanjan University of Medical Sciences, Rafsanjan, IR Iran

**Keywords:** Cytokines, Hepatitis C Virus, Infection

*Dear Editor,*

We have read the article published in your journal written by Sofian et al., entitled “the serum profile of Th1 and Th2 cytokines in treated and non-treated HCV infected individuals” (1). Evidences well documented that the simultaneous elevation of Th1 and Th2 related cytokines during HCV infection may indicate that both (Thl and Th2) cytokine profiles are involved in the pathogenesis of the diseases (2). Moreover, this activated T cell response in HCV infected patients could be regulated by treatment protocols (3). Sofian et al.’s data provides some additional evidence for the involvement of cellular immune response in HCV infections arrest (1). Accordingly, based on the importance of serum profile of Th1 and Th2 cytokines in HCV infected patients, the authors raised the interesting idea that there might be an association between these cytokines and HCV (1).

Authors of the present letter believe that Sofian and co-workers’ results need to be interpreted with more cautious, because possible puzzles and limitations of their early concepts of Th1 and Th2 cytokines need to be reconsidered. It is almost widely accepted that in addition to Th2 cell type, IL-10 is produced by a broad variety of other cell types including activated macrophages as well as B and T regulatory (Treg) lymphocytes (4). Similarly, IL-2 is also reported to be secreted from all lymphoid cell types in one side and is considered as a common growth factor, in an autocrine and paracrine fashion, for all lymphocytes on the other side (5). Therefore, it seems that Sofian et al. cytokines classifications were not accurately defined and need to be reconsidered. Furthermore, authors used the general word "profile" in the title of their article, but they only analyzed two cytokines in Th1 and Th2 classes. Thus, it does not mean that they studied the whole Th1 and Th2 cytokines profiles in the study. Additionally, the authors have not discussed their results properly. Their results have demonstrated that the serum levels of both inflammatory (IFN-γ and IL-2) and anti-inflammatory (IL-4 and IL-10) cytokines were higher in HCV infected patients in comparison with controls (1). It has been established that cytokines are fit in a network and alteration in expression of a cytokine can affect others (6). Hence, the results of Sofial et al. may reveal that HCV induces expression of IFN-γ and IL-2 as inflammatory responses and, subsequently, the inflammatory cytokines induce expression of anti-inflammatory cytokines to regulate immune hypersensitivity (4). On the other hand, treatment of HCV infected patients led to decrease of the cytokines (1). Hence, it could be concluded that the declined cytokines expression may possibly be related to the following two mechanisms: 1) the effect of chemical drugs on the immune responses and 2) The decrease of immune system activation due to the Inhibition of HCV replication.
